# Anesthetic management of right radical nephrectomy and inferior vena cava thrombectomy for giant renal cell carcinoma: a case report

**DOI:** 10.1097/RC9.0000000000001121

**Published:** 2026-10-13

**Authors:** Ayoub Bouaiyda, Mohammed Enaimi, Ilyas Massad, Redouane Ahtil, Mustapha Bensghir

**Affiliations:** Department of Anesthesia and Intensive Care, Military Teaching Hospital Mohammed V, Faculty of Medicine and Pharmacy of Rabat, Mohammed V University, Rabat, Morocco

**Keywords:** anesthesia, case report, massive hemorrhage, radical nephrectomy, renal cell carcinoma, tumor thrombus

## Abstract

**Introduction and importance:**

Renal cell carcinoma (RCC) with tumor thrombus extension into the inferior vena cava (IVC) represents a major surgical and anesthetic challenge because of the high risk of massive hemorrhage, hemodynamic instability, and intraoperative tumor embolism.

**Case presentation:**

We report the case of a 55-year-old man presenting with a large right renal mass measuring 14 × 14 × 13 cm, with a tumor thrombus extending from the right renal vein into the infrahepatic IVC. The patient underwent right radical nephrectomy with caval thrombectomy under general anesthesia and invasive hemodynamic monitoring. Anesthetic management focused on anticipating major blood loss, preventing thromboembolic complications related to thrombus manipulation, ensuring adequate blood product availability, and maintaining perioperative hemodynamic optimization. Intraoperative blood loss was estimated at 3500 mL, which necessitated transfusion of four units of packed red blood cells and four units of fresh frozen plasma. The intraoperative course was favorable, with satisfactory hemodynamic stability throughout the procedure, and the patient was subsequently transferred to the intensive care unit for postoperative monitoring.

**Clinical discussion:**

This case highlights the importance of meticulous multidisciplinary planning and perioperative anesthetic management in patients with locally advanced RCC and an IVC tumor thrombus to minimize potentially fatal hemorrhagic and thromboembolic complications.

**Conclusion:**

In summary, this report demonstrates the anesthesiologist’s critical role in mitigating the severe hemodynamic and hemorrhagic risks of radical nephrectomy with IVC thrombectomy through meticulous, multidisciplinary perioperative planning. Specifically, this case emphasizes three key clinical messages: (1) early multidisciplinary planning is mandatory for complex urologic oncology; (2) continuous invasive hemodynamic monitoring combined with early vasopressor support is crucial to manage abrupt preload reductions during IVC clamping; and (3) proactive transfusion preparedness via a standardized massive transfusion protocol prevents coagulopathy during massive hemorrhage.

## Introduction

Renal cell carcinomas (RCCs) are primary malignant kidney tumors, with venous tumor thrombus extending into the inferior vena cava (IVC) in 4–5% of cases. Surgical resection via radical nephrectomy and caval thrombectomy remains the gold standard treatment. A major perioperative challenge of this procedure lies in the severe risks of massive hemorrhage, hemodynamic instability, and acute tumor embolism. While similar cases have been documented, this specific case is notable for the extreme size of the renal mass (14 × 14 × 13 cm) combined with massive intraoperative blood loss (>3500 mL), and these were successfully managed with advanced goal-directed fluid therapy and vasopressor support in a standard tertiary care setting without the necessity of cardiopulmonary bypass. Given these substantial surgical risks, meticulous multidisciplinary planning and tailored anesthetic management are essential.

HIGHLIGHTS

- A giant renal cell carcinoma with an inferior vena cava (IVC) thrombus poses severe risks of hemorrhage and tumor embolism.
- Massive blood loss requires proactive transfusion and multidisciplinary planning.
- Invasive monitoring ensures hemodynamic stability during abrupt IVC clamping.
- Goal-directed fluid therapy is critical to balance perfusion with hemorrhage control.

Our work has been reported in line with the revised SCARE Guidelines 2025 criteria^[^1^]^.

## Case presentation

A 55-year-old man with no significant past medical history was admitted for surgical management of a large right renal mass that had been discovered during the investigation of persistent right flank pain. The patient was a civil servant of North African descent. His family history was unremarkable, and social history revealed no tobacco or alcohol use. He had no known drug allergies and was not taking any chronic medications.

Preoperative abdominal magnetic resonance imaging revealed a right renal tumor measuring 14 × 14 × 13 cm, occupying almost the entire right kidney, with associated tumor thrombosis of the right renal vein extending into the infrahepatic IVC (Mayo Classification Level II). No supradiaphragmatic extension or distant metastases were identified (Fig. 1). The initial differential diagnosis included a primary retroperitoneal sarcoma or a massive adrenal carcinoma; however, the imaging characteristics strongly indicated RCC with venous extension. The patient was clinically staged as cT3bN0M0.

**Figure 1.**
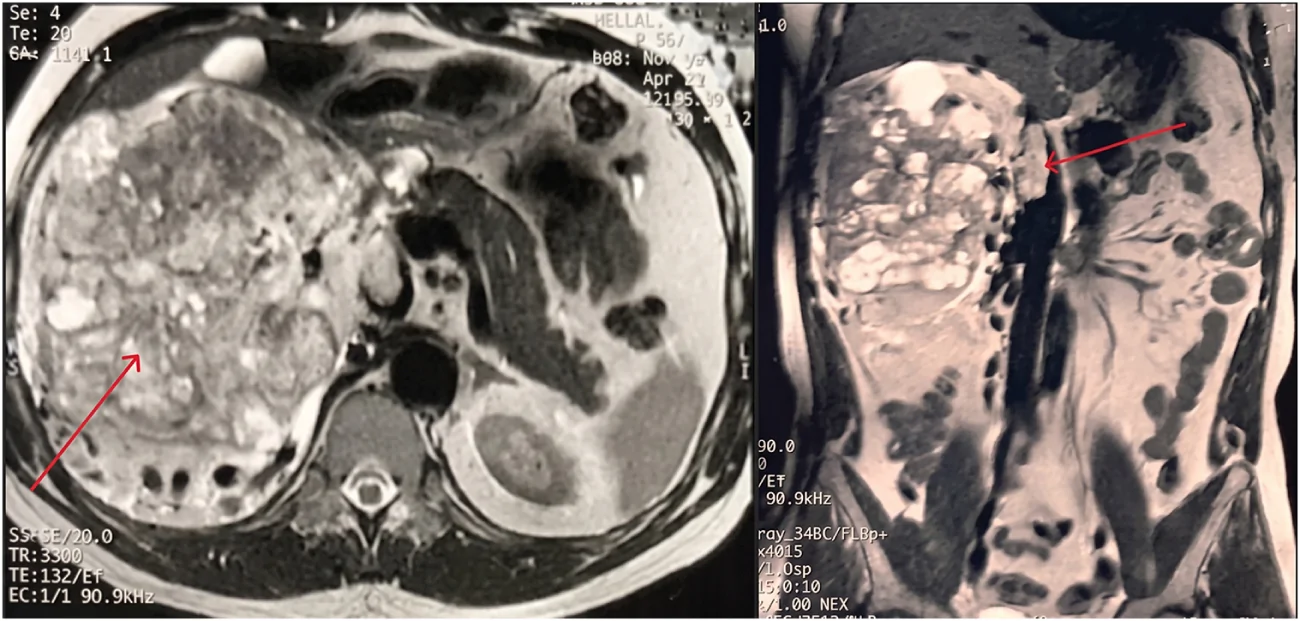
Preoperative magnetic resonance imaging demonstrating a large right renal mass with extension of venous tumor thrombus into the inferior vena cava.

Preoperative clinical evaluation revealed a patient in good general condition (American Society of Anesthesiologists Physical Status II), weighing 82 kg and standing 1.74 m tall. Hemodynamic parameters were stable, including a blood pressure of 120/62 mmHg, a heart rate of 72 beats per minute, and a peripheral oxygen saturation of 100% on room air. Laboratory investigations showed a hemoglobin concentration of 13 g/dL, preserved renal function with a serum creatinine level of 6 mg/L, and no significant coagulation abnormalities.

The patient underwent an extended right radical nephrectomy with IVC thrombectomy, performed under general anesthesia by a senior urologic oncology surgical team. Preoperative optimization included fasting protocols and the administration of prophylactic broad-spectrum antibiotics (cefazolin 2 g IV) 30 minutes prior to incision. The patient was placed in a modified left flank position, and standard chlorhexidine skin preparation was applied. Standard monitoring was supplemented by invasive arterial blood pressure monitoring and central venous catheterization. Anesthesia was induced using propofol, fentanyl, and rocuronium, followed by uneventful endotracheal intubation. Maintenance of anesthesia was achieved with volatile anesthetic agents and opioid analgesia.

The operation was completed without major intraoperative complications (Fig. 2). Surgical isolation was meticulously achieved through sequential cross-clamping of the infrarenal IVC, the contralateral renal vein, and the suprarenal IVC to minimize thromboembolic risk prior to cavotomy. Estimated blood loss was approximately 3500 mL, calculated volumetrically via suction canisters and gravimetrically by weighing surgical sponges. To manage this hemorrhage, our institutional Massive Transfusion Protocol (MTP) was activated, using a targeted 1:1:1 ratio of blood products. This required the transfusion of four units of packed red blood cells and four units of fresh frozen plasma. Continuous hemodynamic monitoring and goal-directed fluid therapy maintained satisfactory circulatory stability throughout the procedure (Fig. 3). A continuous infusion of norepinephrine (ranging from 0.05 to 0.15 µg/kg/min) was used as the primary vasopressor support to maintain a mean arterial pressure >65 mmHg, particularly during the abrupt reduction in venous return associated with IVC clamping.

**Figure 2.**
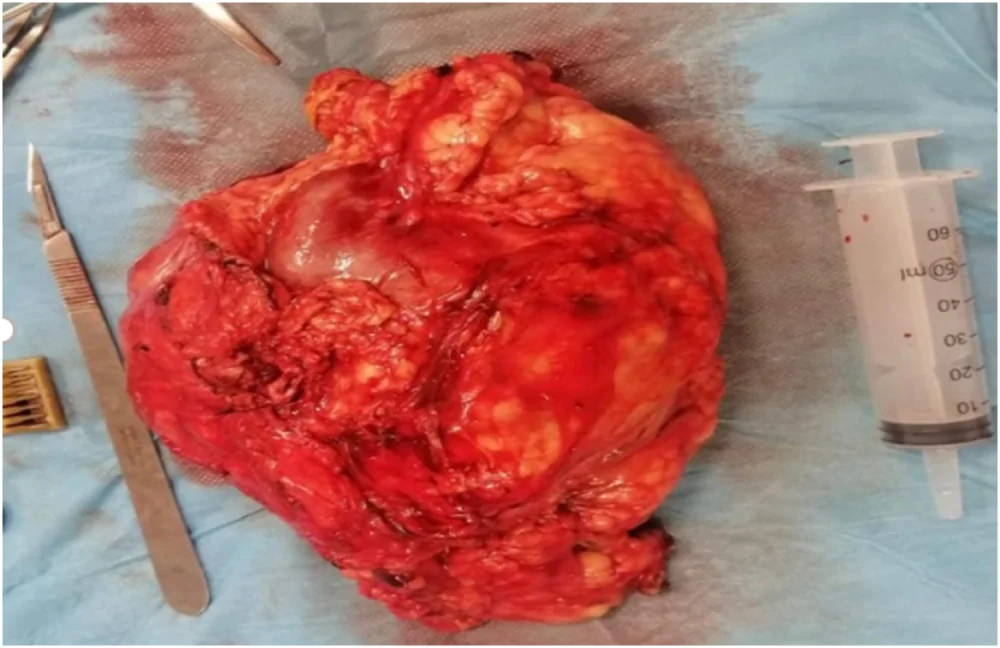
Surgical specimen following a right radical nephrectomy with en bloc resection of the large renal tumor and the caval thrombus.

**Figure 3.**
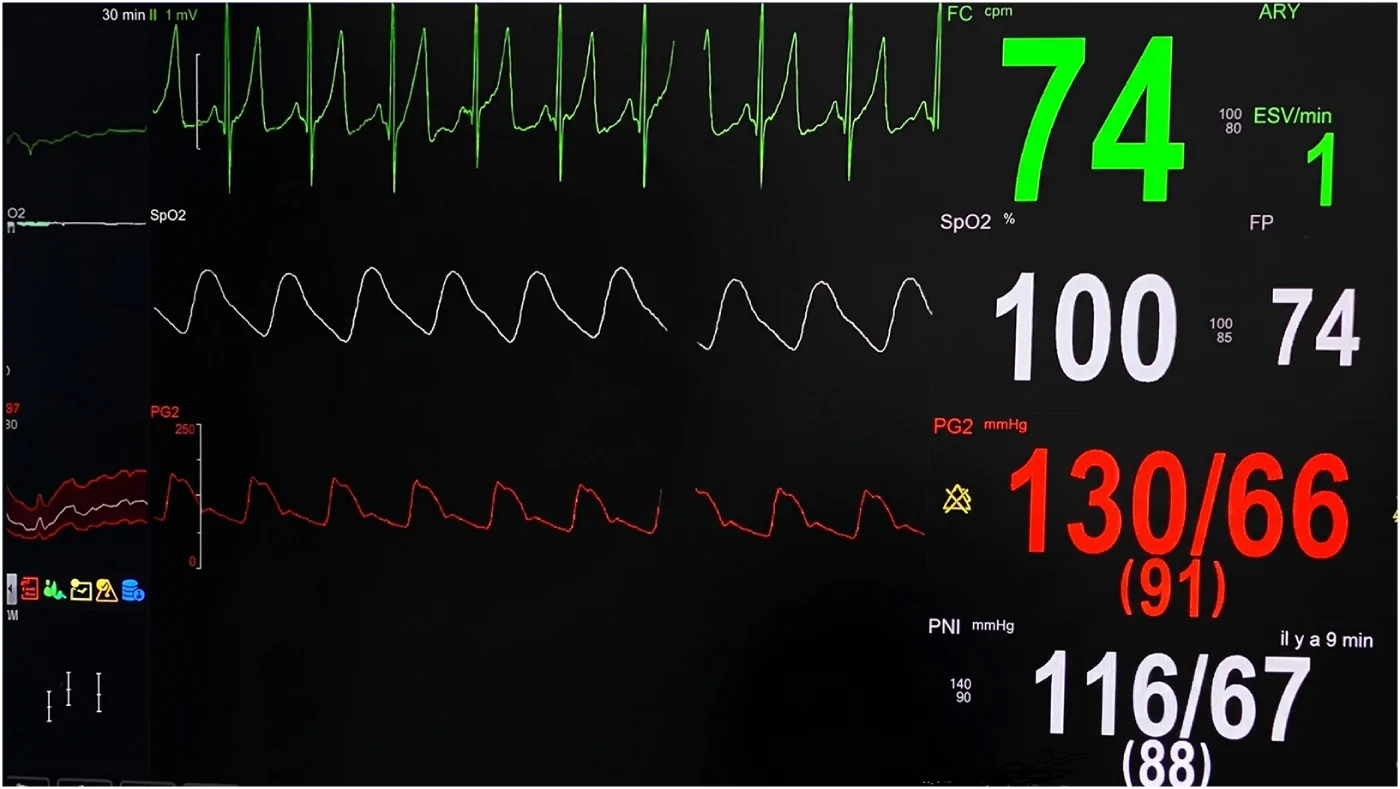
Intraoperative anesthetic monitoring demonstrates stable hemodynamic and respiratory parameters during the procedure.

At the end of the surgery, the patient was transferred to the intensive care unit for close postoperative monitoring, including hemodynamic and laboratory surveillance, given the substantial risks of hemorrhagic and thromboembolic complications associated with caval extension of the renal tumor. Chemical thromboprophylaxis was withheld preoperatively due to bleeding risks, but was initiated 24 hours postoperatively following the confirmation of hemostasis.

## Timeline

A summary of the patient’s clinical course is provided in Table 1.

**Table 1:** A summary of the patient’s clinical course.

Phase	Event description
Initial presentation	Admitted for persistent right flank pain; subsequent investigation revealed a right renal mass.
Diagnostic assessment	MRI confirmed a 14 × 14 × 13 cm primary renal mass with infrahepatic IVC tumor thrombus (cT3bN0M0, Mayo Level II).
Surgical intervention	Right radical nephrectomy and IVC thrombectomy performed. EBL 3500 mL managed successfully with MTP and continuous vasopressor support.
Postoperative course	ICU admission for 48 hours for hemodynamic and coagulation surveillance; stepped down to the general urology ward.
Discharge	Discharged on postoperative day 7 without major complications (Clavien–Dindo Grade II for blood transfusion).
Follow-up	Six-month postoperative surveillance revealed excellent functional recovery and no clinical or radiological evidence of disease recurrence.

EBL, estimated blood loss; MRI, magnetic resonance imaging; MTP, massive transfusion protocol.

## Discussion

RCC accounts for approximately 2–3% of all adult malignancies and represents the most common primary malignant tumor of the kidney. Venous tumor extension occurs in 4–10% of cases, while involvement of the IVC is observed in approximately 4–5% of patients at the time of diagnosis^[^2,3^]^. Despite its locally advanced nature, the presence of a venous tumor thrombus is not considered metastatic disease, and complete surgical resection remains the gold-standard treatment whenever technically feasible^[^3–5^]^. In the present case, the patient presented with a giant right renal mass associated with extension of the tumor thrombus into the infrahepatic IVC (Mayo Level II), requiring radical nephrectomy and caval thrombectomy.

Radical nephrectomy with IVC thrombectomy is among the most technically demanding procedures in urologic oncology. Surgical complexity depends on the cranial extent of the thrombus, its adherence to the caval wall, and the presence of suprahepatic or intracardiac extension^[^4,6^]^. Current European Association of Urology guidelines recommend complete surgical excision in appropriately selected patients, as this approach offers the best opportunity for long-term oncologic control and survival^[^3^]^. Several large surgical series have reported 5-year cancer-specific survival rates exceeding 50% in patients undergoing complete resection in the absence of distant metastatic disease^[^5,7^]^. The complexity of managing extreme vascular involvement is further underscored in the literature; for instance, Ghanney *et al*^[^8^]^ demonstrated that even in highly specialized scenarios, such as pregnancy, tumor thrombus extending to the right atrium poses extraordinary multidisciplinary challenges. Similarly, Murtaza *et al*^[^9^]^ highlighted that navigating the complexity of these cases hinges fundamentally on seamless anesthetic and surgical communication.

From an anesthetic perspective, the risk of massive intraoperative hemorrhage remains the primary concern. Blood loss during nephrectomy with caval thrombectomy is highly variable and may reach several liters depending on thrombus level, vascular involvement, and surgical complexity^[^6,10^]^. In a multicenter study, Abel *et al* reported substantial transfusion requirements in nearly half of patients undergoing surgery for RCC with venous tumor thrombus^[^6^]^. In our patient, estimated blood loss reached 3500 mL, necessitating a transfusion of four units of packed red blood cells and four units of fresh frozen plasma. These findings underscore the importance of meticulous preoperative blood product preparation, the use of standardized MTP protocols, reliable large-bore vascular access, invasive hemodynamic monitoring, and prompt correction of hemodynamic disturbances during surgery^[^6,11^]^.

Another major anesthetic challenge is the risk of thromboembolic complications. Manipulation of the tumor thrombus may result in fragmentation or migration into the pulmonary circulation, leading to acute tumor embolism, a rare but potentially catastrophic event associated with high mortality^[^12,13^]^. Consequently, close coordination between anesthesiologists and surgeons is essential during critical stages of the operation, particularly renal mobilization, vascular clamping, and thrombus extraction. Intraoperative transesophageal echocardiography (TEE) has been advocated by several authors, especially in patients with extensive thrombi, because it allows real-time assessment of thrombus position and facilitates early detection of embolic migration^[^13–15^]^. Although TEE was not used in our case, it remains a valuable adjunct in selected high-risk patients.

Significant hemodynamic alterations may also occur during IVC clamping. Abrupt reduction in venous return can result in marked decreases in preload, cardiac output, and systemic arterial pressure^[^11,14^]^. Continuous invasive hemodynamic monitoring, individualized goal-directed fluid therapy, and early vasopressor support (such as the continuous norepinephrine infusion utilized in our patient) are therefore essential to maintain adequate organ perfusion while avoiding excessive fluid administration that may exacerbate surgical bleeding^[^11,16^]^. This delicate balance between hemodynamic optimization and hemorrhage control represents a cornerstone of anesthetic management during caval thrombectomy.

The favorable outcomes achieved in recent decades are largely attributable to a multidisciplinary approach involving urologists, anesthesiologists, vascular surgeons, radiologists, intensivists, and transfusion medicine specialists^[^6,15^]^. Advances in perioperative monitoring, surgical techniques, blood conservation strategies, and postoperative critical care have significantly reduced perioperative morbidity and mortality in specialized centers^[^6,7^]^. Our case highlights the pivotal role of comprehensive preoperative assessment, anticipation of hemorrhagic and thromboembolic complications, and tailored anesthetic management in optimizing outcomes for patients undergoing radical nephrectomy with IVC thrombectomy for locally advanced RCC.

### Take-away lessons

* **Proactive resource allocation**: Massive hemorrhage must be anticipated. Implementing an institutional MTP protocol and securing cross-matched blood products preoperatively are vital.

* **Hemodynamic anticipation**: IVC cross-clamping induces profound preload deficits. Invasive arterial monitoring and continuous vasopressor infusions should be initiated proactively rather than reactively.

* **Collaborative communication**: Surgical milestones (tumor mobilization, clamping, and thrombectomy) require explicit closed-loop communication between the surgical and anesthesia teams to mitigate embolic events and hemodynamic collapse.

### Strengths and limitations

A major strength of this report is the detailed documentation of both surgical steps and specific anesthetic interventions (MTP utilization, vasopressor choices, and fluid strategy) during a procedure with massive blood loss. A limitation of this case is the absence of intraoperative TEE, which, while not utilized here, is increasingly considered a standard of care for visualizing thrombus mobility and cardiac function during complex thrombectomies.

## Conclusion

Radical nephrectomy with IVC thrombectomy for RCC with venous tumor extension remains a highly challenging surgical procedure associated with substantial risks, including massive hemorrhage, hemodynamic instability, and tumor embolization. Successful perioperative management relies on meticulous preoperative planning, invasive hemodynamic monitoring, adequate transfusion preparedness, and close multidisciplinary collaboration. This case emphasizes the crucial role of the anesthesiologist in anticipating and managing these potentially life-threatening complications, thereby contributing significantly to favorable surgical outcomes.

## Data Availability

The data that support the findings of this study are available from the corresponding author upon reasonable request.

## Declaration on the use of Generative AI

In the preparation of this manuscript, a generative AI tool “ChatGPT” was employed exclusively to assist with grammar and syntax correction. The tool was used during the drafting phase in July 2026. The application of this tool did not influence the research design, data analysis, or interpretation of results. All scientific content, insights, and conclusions presented herein are solely the responsibility of the authors. Every AI-generated correction was carefully reviewed and validated line-by-line by the authors against the original source material to ensure that the integrity and accuracy of the content were maintained.
